# Animal Quinine

**Published:** 1870-09

**Authors:** 


### Animal Quinine.

Mr. Jabez Hogg, in his pamphlet on cataract, informs us that "the extraordinary rapidity with which the nutritive processes are carried on in the body is beautifully exemplified under certain conditions in the refracting media of the eyes. Dr. Bence Jones found that a small dose of lithium in the course of a few minutes passed through the circulation into every part of the body, even into those parts most distant from the central blood supply. The living eye gives the earliest indication of the presence of this remedy. When sulphate of quinine is administered like lithium, and other substances, it rapidly passes from the blood into the textures of the body. Within a quarter of an hour, increased fluorescence is noticed in the nervous texture, in the aqueous humor and in the lens. This observation led to the discovery that a substance resembling quinine is always present in the animal body. It is believed that this animal quinine is descended from albumen, and doubtless is an alkaline fluorescent substance of the utmost importance in the animal economy."

